# Postictal Petechiae as a Cutaneous Manifestation Following Generalized Tonic-Clonic Seizures

**DOI:** 10.7759/cureus.22437

**Published:** 2022-02-21

**Authors:** Eric D Schadler, Matthew Friedland, Jennifer Mancuso

**Affiliations:** 1 Department of Dermatology, University of Michigan, Ann Arbor, USA

**Keywords:** cutaneous manifestation, uncommon rash, generalized tonic-clonic seizures, generalized seizure, petechiae, postictal petechiae

## Abstract

Identifying cutaneous manifestations associated with systemic diseases is a crucial task for dermatologists and other providers in the outpatient and inpatient settings. Here, we present a rare case of postictal petechiae occurring after a generalized tonic-clonic seizure in a patient with poorly controlled epilepsy. This case illustrates a unique and underrecognized entity that may serve as the only cutaneous clue to assist in the diagnosis of recent seizure activity.

## Introduction

Various cutaneous manifestations have been described following episodes of generalized tonic-clonic seizures (GTCs), including ecchymoses, abrasions, lacerations, and oral lesions. Postictal petechiae, also referred to as postictal thoracocervicofacial purpura by some neurologists, is a rare and underreported finding associated with GTCs that has been documented in the neurology literature, yet has been undescribed by dermatologists [1-4]. Knowledge of this cutaneous entity is important for dermatologists and other healthcare providers to recognize because lesions can be distressing to patients, can be mistaken for a drug eruption, and, on occasion, can be an isolated cutaneous clue to seizure activity. Here, we present a case of postictal petechiae occurring in a young man after a witnessed GTC. Patient consent was obtained for the use of all images.

## Case presentation

A 20-year-old man with a history concerning for juvenile myoclonic epilepsy, managed with levetiracetam 1,000 mg twice daily, presented to the emergency department for evaluation following a witnessed GTC seizure. The breakthrough seizure lasted approximately five minutes and resulted in a ground-level fall. Characteristic tonic-clonic muscle contraction with rhythmic twitching was described by the present family members, followed by a transient period of confusion. His medical history was otherwise unremarkable.

Vital signs upon arrival to the hospital were within normal limits. The patient’s neurologic examination was unremarkable without focal deficits, and a CT scan of the head and neck revealed no acute abnormalities. A review of the systems was positive for residual fatigue. Lab work revealed leukocytosis and cannabis on the urine drug screen. Notably, platelet count (297 K/µL) and comprehensive metabolic panel were both unremarkable.

**Figure 1:**
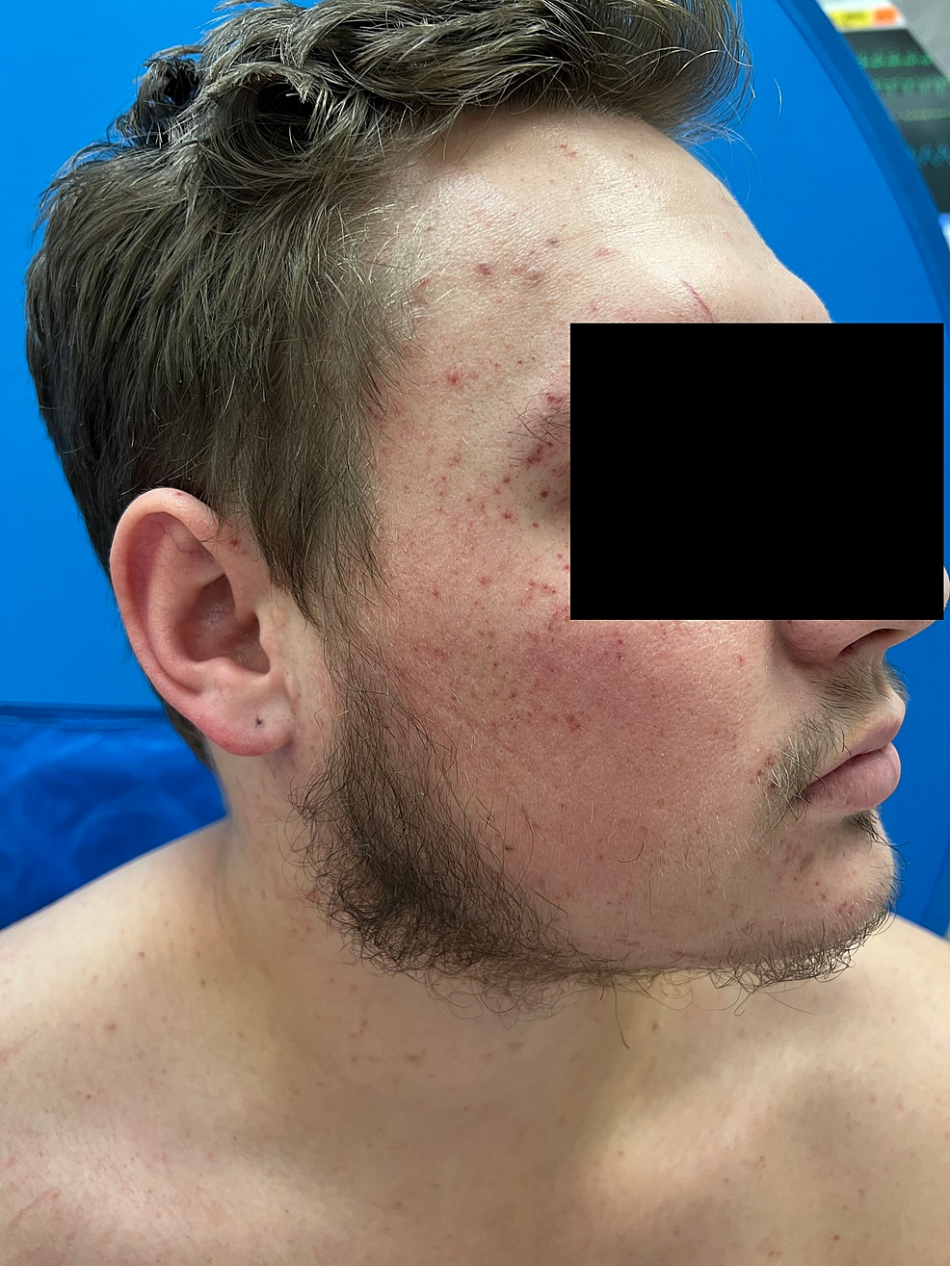
Periorbital petechiae extending onto the forehead, temple, and cheek.

**Figure 2:**
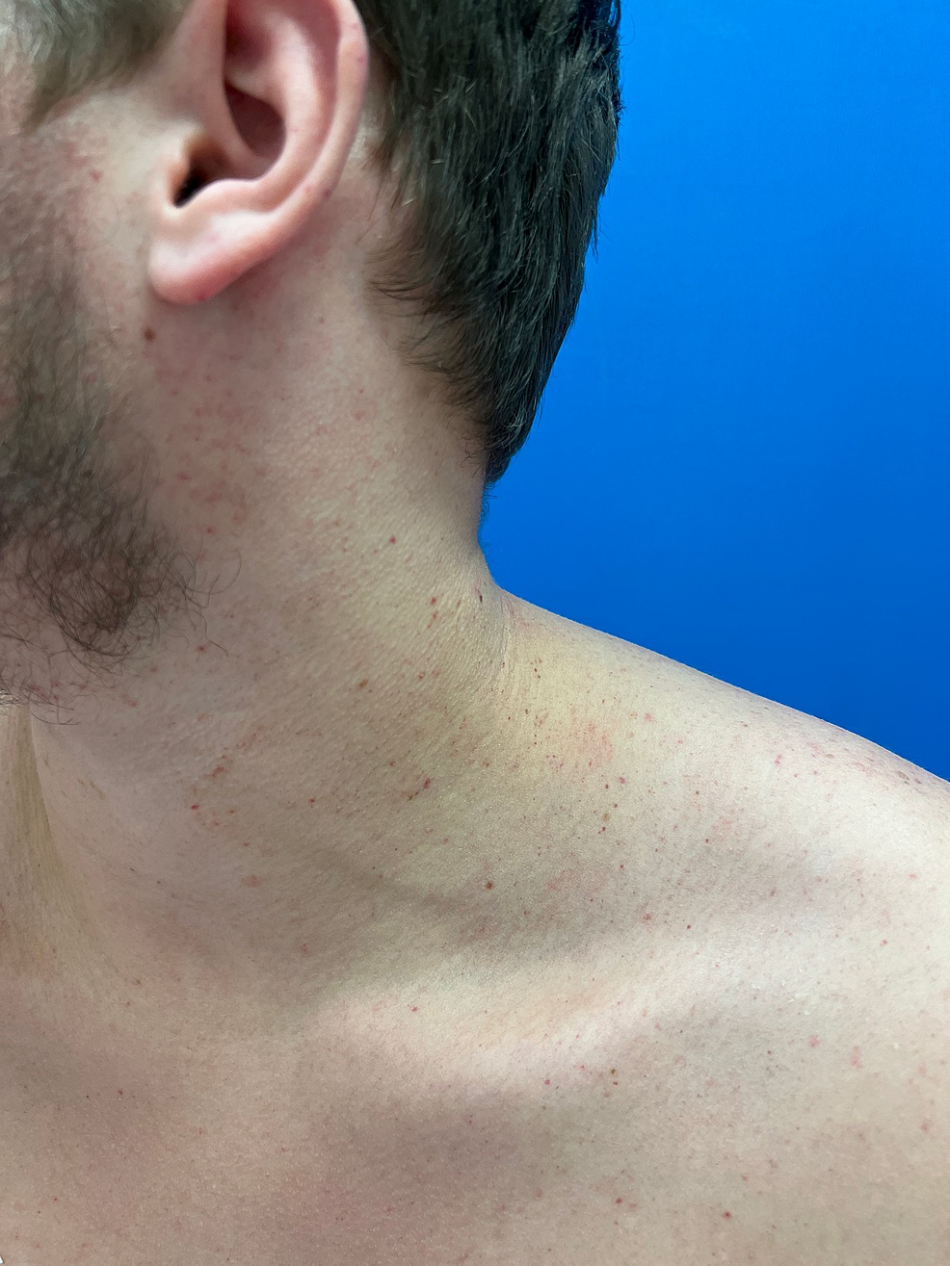
Petechiae seen on the left ear, lateral neck, and shoulder.

Dermatology was consulted after initial evaluation for the presence of a new rash temporally associated with his seizure, with concern from the primary team for a drug-induced rash. On examination, many non-palpable, non-blanching petechiae were distributed on the periorbital skin, eyelids, cheeks, neck, and back (Figures 1, 2). Lesions were non-inflammatory in appearance. With exception of ecchymoses present on the lower extremity, his skin and oral examinations were unremarkable. Unfortunately, a fundoscopic examination was not performed during his time in the hospital to examine for the involvement of the retina. The patient reported similar appearing rashes that had occurred in the past after GTCs which self-resolved after a few days. Ultimately, after eliminating other causes of petechiae through history and lab work, a diagnosis of postictal petechiae was made. No further treatments were recommended from a dermatologic perspective, and neurology follow-up was arranged to address the breakthrough seizure activity.

## Discussion

Petechiae are less than 4 mm red to purple macules that develop from capillary bleeding beneath the skin surface. They may occur due to platelet abnormalities, including those related to thrombocytopenia (e.g., idiopathic autoimmune thrombocytopenia, thrombotic thrombocytopenia, disseminated intravascular coagulation, drug-related thrombocytopenia, etc.), impaired function (e.g., hereditary platelet defects, drug-related inhibition, monoclonal gammopathy, thrombocytosis, etc.), and non-platelet etiologies such as trauma, inflammatory dermatoses, and spiking elevations of vascular pressure (coughing, vomiting, childbirth, blood pressure measurements, etc.) [5].

Although the exact pathophysiology of postictal petechiae is not entirely understood, multiple mechanisms have been proposed [2,3]. One suggestion, favored by the authors in this case, is mechanical damage resulting from intense muscular contraction during the tonic-clonic phase creating a prolonged, unintentional Valsalva maneuver. As with cases of petechiae resulting from protracted vomiting or paroxysmal coughing fits, the Valsalva maneuver increases intra-abdominal pressure subsequently decreasing cardiac preload and increasing peripheral venous pressures. Rupture of thin-walled capillaries and post-capillary venules because of this pressure leads to the formation of petechiae. An alternative proposition discussed the release of interleukin-6 (IL-6) following seizure activity, which may modify platelet function [2,6,7]. IL-6 is an inflammatory cytokine that mediates activity through binding the IL-6 receptor or β-receptor glycoprotein 130 (gp130). Platelets express gp130 on their membranes and upon activation may lead to the development of vessel inflammation and thrombogenesis [7]. Clinically, the lack of inflammation that would present as blanching erythema surrounding the petechiae argues against this mechanism. Lastly, it has been proposed that neuronal release of vasoactive mediators following a seizure may damage capillaries resulting in the lesions on the face [2]. The localized distribution of postictal petechiae argues against these last two suggestions, which would likely favor dependent areas.

Postictal petechiae is a diagnosis of exclusion after ruling out more sinister differential diagnoses mentioned above. Workup of patients with this condition should include a thorough medical history, physical examination, and basic labs, including a complete blood count with differential, coagulation studies (prothrombin time and partial thromboplastin time), and metabolic panel. In patients with signs or symptoms suggestive of active infection, additional infectious workup should be performed. Depending on the clinical scenario, life-threatening infections, such as Rocky Mountain spotted fever, meningococcemia, or infectious endocarditis, and more innocuous infections, such as cytomegalovirus or Epstein-Barr virus, should be considered. A dermatologist evaluation would also help to differentiate petechiae from a morbilliform drug eruption, which is frequently a concern given the risk of severe cutaneous adverse reactions from antiepileptics.

## Conclusions

Awareness of postictal petechiae as a benign cutaneous manifestation of seizures is important for dermatologists and healthcare providers to reassure patients, family members, or other hospital staff. In the appropriate clinical setting, a rapid diagnosis can be made to save patients undue anxiety; however, this first requires knowledge of this entity.
